# Safe Debonding of Fixed Appliances: A Comparison of Traditional Techniques and LODI Devices on Different Bracket Types in Terms of Enamel Cracks, Site of Bond Failure, and Bracket Reusability

**DOI:** 10.3390/ijerph181910267

**Published:** 2021-09-29

**Authors:** Marta Gibas-Stanek, Małgorzata Pihut

**Affiliations:** 1 Department of Orthodontics, Dental Institute, Faculty of Medicine, Jagiellonian University Medical College, 31-155 Krakow, Poland; 2 Department of Prosthodontics, Dental Institute, Faculty of Medicine, Jagiellonian University Medical College, 31-155 Krakow, Poland; pihut_m@poczta.onet.pl

**Keywords:** orthodontic brackets, debonding, debonding pliers

## Abstract

The objective of this study was to compare the effects of the debonding of three different bracket types by means of three popular debonding methods. A total of 180 human third molars was divided into six groups, consisting of 20 teeth each. Three bracket types were bonded to the enamel (metal brackets with an integral base and a foil mesh base, and ceramic brackets) and three methods of bracket debonding were employed (bracket removal pliers, Weingart pliers, and Lift-Off Debonding Instrument). The samples were examined with scanning electron microscopy to assess the number of enamel cracks, measure the area of adhesive remaining on the enamel, and calculate the adhesive remnant index (ARI). There were no statistically significant differences between the groups in terms of the number of enamel cracks after bracket debonding. The amount of adhesive remaining on the teeth after the brackets were removed was significantly different between the groups. LODI and Weingart pliers are considered to be the safest methods of debonding brackets with an integral base, while LODI is the best tool for brackets with foil mesh. Bracket removal pliers are considered to be the preferred method for ceramic bracket debonding.

## 1. Introduction

The success of orthodontic treatment with a fixed appliance largely depends on the stable retention of its elements on the enamel surface throughout therapy. Apart from the physical and chemical properties of the adhesive material, one of the factors responsible for bond strength is the construction of the bracket base, which is the architecture that provides mechanical retention for the adhesive used. The objective of bracket production is to obtain the flow of material in the irregularity of the base. The most popular methods are the use of a retention mesh soldered to a metal base, which is the case with two-piece brackets, and the use of retention undercuts on the surface of the bracket facing the enamel (a method typically used for one-piece metal brackets and composite, and ceramic brackets).

Manufacturers of fixed appliances are making efforts to increase the strength of the bonds between brackets and enamel by subjecting them to chemical etching or laser treatment, but there are studies showing that excessive adhesion can lead to irreversible damage to the tooth’s surface during debonding [1,2,3,4]. The safest method to remove a fixed appliance is to break the mechanical connection between the bracket base and the adhesive material [5]. Nevertheless, in practice, damage occurs not only at the enamel–adhesive material interface and within the adhesive material, but also within the enamel itself. Data from previous research and personal experience indicate that there is a relationship between the bracket base structure and the amount of force necessary to detach it, which suggests the existence of superior forces in brackets with anchor pylons and ceramic brackets compared to mesh brackets [6,7]. A study that compared the shear force necessary to detach a bracket with a retention mesh (3M Victory Series, Orthodontic Design and Production, Monrovia, CA, USA) and a bracket with anchor pylons (Cannon System, Orthodontic Design and Production, Stratford, CT, USA) showed average forces of 12.53 MPa and 18.93 MPa, respectively [8]. Reddy and Rohit demonstrated that the mean shear bond strength of ceramic brackets was 20.68 MPa and that of metal brackets was 12.15 MPa [9].

In vitro experimental studies have reported that the shear bond strength recorded during the debonding of orthodontic brackets was less than the tensile bond strength, which means that not only the type of bracket base but also the type of force applied is important when disassembling a fixed appliance [10,11].

Although different ultrasonic and electrothermal debonding methods have been proposed to optimize the process of bracket removal [7,12], the most popular techniques used in everyday practice, due to availability and affordability, are mechanical debonding with a lift-off debonding instrument (LODI, ^®^3M Unitek, St. Paul, MN, USA), with bracket removal pliers, or with Weingart pliers. These instruments exert different types of forces (shear force and tension force) on different parts of the bracket (base or wings) and can potentially cause various deformations of the bracket structure, which can lead to bond failure on four different levels: the bracket–adhesive interface, within the adhesive, the adhesive–enamel interface, and within the enamel [13].

Due to the availability of orthodontic brackets of various designs on the market, it seems reasonable to try to define the method of debonding that is the safest for a given type of retention base. The aim of this study was to compare three methods of debonding in three different bracket systems.

## 2. Materials and Methods

### 2.1. Sample Preparation

For the purpose of this study, 193 freshly extracted (for orthodontic reasons) human third molars were collected from patients who gave consent for their teeth to be used in the experiment. The criteria for including samples in the study were: no demineralization or hypomineralization, no history of any pretreatment with chemical agents, no restorations, no cracks during extraction, and no caries.

The sample size was estimated using a sample size calculator. According to the results, a sample size of 129 was required to detect differences with a 5% margin of error and a 95% confidence level (population size: 193). The final number of teeth included in the study was higher than the number calculated.

**Figure 1:**
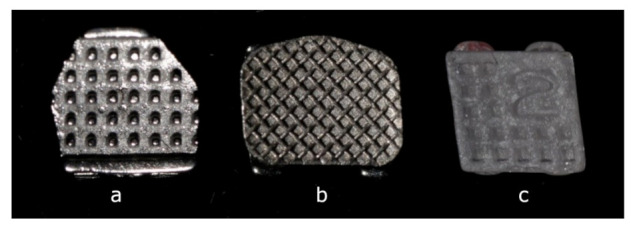
Types of brackets tested in the study: (**a**) Cannon System metal bracket (bracket base with anchor pylons), (**b**) Victory Series 3M metal bracket (bracket base with foil mesh), (**c**) Backlight ceramic bracket (bracket base with retention undercuts).

The teeth were cleansed of soft tissue and stored in tap water at room temperature. All of the teeth were assessed using an optical microscope (Leica Wild m650) at 16× magnification, and the transillumination method was used to locate the flattest area on the buccal or lingual tooth surface without visible cracks in the enamel. Ultimately, 180 samples were included in the analysis. Subsequently, the selected area was marked with a waterproof marker. The samples were randomly assigned to three groups (60 samples in each group), and three types of brackets with 0.018-inch slots were bonded to the enamel (Figure 1):

(a) Cannon Ultra metal brackets for upper central incisors (60 pieces), chosen as representatives of one-piece metal brackets with an anchor pylons base. The Cannon Ultra bracket is a twin-slot bracket that combines the properties of a standard edgewise bracket with the low friction properties related to the Begg technique. It consists of two occlusal tie wings and one gingival tie wing.
(b) Victory Series metal brackets (3M Unitek) for upper central incisors (60 pieces), chosen as representatives of a “standard” edgewise type of bracket of good quality with a foil mesh base.
(c) Backlight ceramic brackets for upper lateral incisors (60 pieces), chosen as representatives of esthetic brackets with retention undercuts. Brackets for lateral incisors were chosen due to the correspondence between their size and the dimensions of the metal bracket for central incisors (in order to eliminate the influence of the size of the bracket base on the bond strength).

The brackets for the incisors proved to fit neatly to the buccal and lingual surfaces of the third molars. All the brackets were tested by manually pulling a 0.018 × 0.025-inch stainless steel archwire (American Orthodontics, Sheboygan, WI, USA) through a slot, and no slot distortions were detected at baseline.

### 2.2. Bonding Procedure

The selected surfaces of all the specimens were polished with a mixture of water and fluoride-free pumice in a rubber prophylactic cup for 10 s, rinsed with water spray for 10 s, and subsequently dried with air from a standard triple spray.

The tooth surfaces were etched with 37% phosphoric acid gel applied to the buccal surface of each tooth for 30 s. The teeth were then rinsed with water spray for 30 s and air dried for 20 s. All the brackets were bonded to the prepared tooth surfaces using the Transbond XT adhesive system (3M Unitek), in accordance with the manufacturer’s instructions.

The brackets were then properly positioned on the teeth using bracket tweezers and finally pressed onto the tooth surface. Excess adhesive was removed with a sharp probe, and the brackets were subsequently light-cured for 40 s (10 s on each side of the bracket). The light source came from an Ortholux Luminous Curing Light (3M Unitec, Monrovia, CA, USA) with a wavelength of 430–480 nm and a minimum output light intensity of 1000 mW/cm^2^.

### 2.3. Debonding Procedure

After storage for 24 h in tap water at room temperature, the samples from the three groups were randomly divided into nine subgroups (20 samples in each group), and three methods of bracket debonding were employed in the standardized manner:

1. Bracket removal pliers (CHIFA, KP-013-135-PMK) were placed occluso-gingivally between the bracket base and the tooth surface and gently squeezed; due to the long gingival tie wings in the case of the Cannon brackets, the pliers were placed mesio-gingivally and disto-incisally (obliquely).
2. Weingart pliers (CHIFA, KP-039-140-HPMK) were placed mesio-distally and used to squeeze the bracket wings.
3. A LODI metal hanger of was positioned under the gingival tie wing of the Victory and Backlight brackets, and under the occlusal tie wing in the case of the Cannon brackets, and the instrument was allowed to rest on the tooth. The compression of the handles caused the bracket to lift off upon the application of a pulling force.

All the bonding and debonding procedures were managed by the same operator (M.G.-S.) with eight years of experience in orthodontics.

### 2.4. Measurements

**Figure 2:**
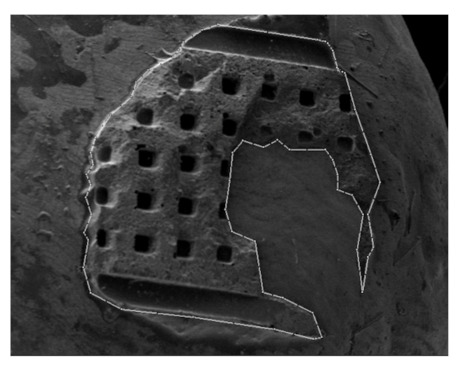
Scanning electron microscopy image (JEOL JSM5410, 15× magnification) of the area of adhesive remaining on the enamel, outlined and calculated with CAD Rysunek 3.48 software.

After debonding, the samples were examined with scanning electron microscopy (SEM) (JEOL JSM5410) at the Department of Cell Biology and Imaging (Institute of Zoology and Biomedical Research, Krakow, Poland), using 15× magnification to assess the number of enamel cracks. The area of adhesive remaining on the enamel was calculated using CAD Rysunek 3.48 software (Figure 2).

The results obtained in square centimeters were divided by the surface area of the bracket base to convert them to percentages. Furthermore, the adhesive remnant index (ARI) established by Artun and Bergland [14] was used to classify the enamel surface after debonding according to the following scores:

Score 0, no composite resin left on the tooth;
Score 1, less than half of the composite resin left on the tooth;
Score 2, more than half of the composite resin left on the tooth;
Score 3, all of the composite resin left on the tooth with a distinct impression of the bracket base.

The debonded brackets were assessed to determine their reusability. First, the base, slot, and tie wings of the brackets were visually inspected and compared to new brackets of the same type. Brackets with visible damage were eliminated from further testing. Brackets without visual damage were tested by manually pulling them through the 0.018 × 0.025-inch stainless steel archwire to detect any signs of slot deformation, such as inhibition of the insertion of the archwire, crepitation, or deadlock. The test was repeated twice by the same investigator (M.G.-S.), and the brackets were classified as reusable if they passed both tests.

### 2.5. Statistical Analysis

The comparisons of the quantitative variables within groups were conducted with the Kruskal-Wallis test. Dunn’s post hoc test was used to determine individual points of significant difference between the groups. The comparisons of the qualitative variables in the groups were conducted using the chi-squared test (with Yates’ correction) or with Fisher’s exact test (when low expected values occurred). All the analyses were conducted at a 0.05 level of significance. R software version 4.0.2 was used.

The study was approved by the bioethics committee of Jagiellonian University (number 1072.6120.139.2018).

## 3. Results

The results of this investigation revealed that six out of 180 specimens featured enamel cracks after bracket debonding; the detailed results are presented in Table 1. Fisher’s exact test showed that there were no statistically significant differences between the groups.

Table 2 shows the percentage of adhesive remaining on the enamel’s surface after the debonding of the ceramic brackets, the metal brackets with anchor pylons, and the metal brackets with foil mesh using three different methods. The Dunn test showed that the amount of adhesive remaining on the teeth after the removal of the Backlight brackets with Weingart pliers (56.85 ± 30.98%) was significantly lower compared to when LODI (86.83 ± 6.76%) and bracket removal pliers (84.36 ± 19.74%) (*p* = 0.01) were used. In the case of the Cannon brackets debonded with the bracket removal pliers, a mean of 25.46 ± 18.36% of the adhesive remained on the teeth, while there was significantly more adhesive in the cases of the LODI and Weingart pliers (70.75 ± 33.75% and 61.58 ± 30.18%, respectively) (*p* < 0.01). The Victory brackets debonded with LODI left significantly more adhesive (73.22 ± 28.75%) compared to the Weingart pliers group (34.21 ± 40.93%) (*p* = 0.017).

Table 3 presents the comparisons according to the type of bracket used. After debonding with debonding pliers, there was significantly more adhesive on the teeth’s surfaces in the case of the Backlight brackets than in the case of the Victory brackets. The lowest amount remained after the removal of the Cannon brackets (*p* < 0.01). By contrast, the Cannon brackets debonded using Weingart pliers left significantly more adhesive than the Victory brackets that were detached using this method (*p* = 0.036). In the groups that were debonded with LODI, there were no statistically significant differences between the three types of brackets tested in the study (*p* = 0.852).

Fisher’s exact test was used to compare the ARI scores between the groups (Table 4 and Table 5). The results mostly confirmed the outcomes of the tests performed on the data obtained from the surface area measurements. The only difference was found in the LODI group, in which, according to the ARI comparison, the highest values were present in the Backlight group and the lowest values were present in the Victory group (*p* = 0.03). In the surface measurements, there were no significant differences between the groups. In the case of the comparison of the ARI scores between groups according to the debonding method, there was no significant differences in the ARI scores of the Cannon group (*p* = 0.06), while in the surface area measurements, the values in the LODI and Weingart groups were significantly higher than in the bracket-removal-with-pliers group (*p* < 0.01).

All the ceramic brackets removed with debonding pliers, 95% of those debonded with Weingart pliers, and 90% of those debonded with LODI were structurally intact, but the differences were statistically insignificant. All of the Cannon brackets that were removed with debonding pliers and LODI were classified as reusable, while 80% of the Cannon brackets removed with the Weingart pliers were damaged.

The differences were also statistically significant in the case of the Victory brackets: 90% of the brackets from the Weingart pliers group and 55% from the debonding pliers group were structurally distorted, but all the brackets debonded with LODI remained intact. The detailed results are presented in Table 6.

## 4. Discussion

Although human premolars are frequently used in adhesion tests in orthodontics, third molars are a suitable alternative due to their large, relatively flat areas of enamel and better accessibility [15]. The teeth selected for this study were carefully evaluated macroscopically and microscopically to achieve the most comparable conditions.

The brackets were debonded after 24 h of storage to obtain maximum bond strength [16], and the tooth surfaces were evaluated under SEM to precisely assess the topography of the enamel and the amount of adhesive remaining on each tooth. Since it is accepted that shear bond strength differs from tensile bond strength (shear bond strength is greater than tensile bond strength), this study aimed to test various methods of debonding. The bracket removal pliers represented shear force, the LODI represented tensile force, and the Weingart pliers represented squeezing force [11,17].

While it was previously demonstrated that anchor pylons bases provided greater retention of adhesive compared to the commonly used foil mesh base [8], and that the debonding of the anchor pylons brackets caused more iatrogenic damage to the enamel surface [6], the results of this study did not support this statement. The differences in the number of enamel cracks were not statistically significant between the groups and, surprisingly, the Cannon brackets group was the only group without enamel cracks visible in the magnification selected for the purpose of the study. The inconsistent results can probably be explained by different research methodologies. The teeth selected for this study showed intact enamel, while Ahangar Atashi et al. measured an increase in the length of existing cracks. As the authors explain, this might be attributed to the presence of prior enamel cracks before bonding that acted as stress accumulation sites that could have led to stress-induced damage [6].

The percentage of enamel cracks in the ceramic brackets groups (5.0%) in this study did not differ significantly from that observed in the metal brackets groups (2.5%). Habibi et al. [18] obtained similar results and reported that the risk of enamel damage when debonding ceramic brackets was not greater than the risk when debonding metal brackets. The authors of another study [5], who evaluated debonded brackets using backscattered SEM with dispersive x-ray spectroscopy to detect the presence of enamel on the base, reported that almost 1/3 of ceramic brackets debonded with debonding pliers and 13.3% of metal brackets with a mesh base debonded with LODI showed enamel on the surface. What is clinically relevant is that the percentage of enamel coverage was high in only a few cases and, as the authors suggested, any defects could have been related to the lower quality of enamel to which the brackets had been bonded. Since it is commonly believed that the site of failure is closely related to the risk of enamel cracking, we aimed to determine the amount of adhesive remaining on the tooth’s surface after debonding [5]. According to Artun and Bergland [14], the enamel is protected if the line of fracture is located exclusively within the adhesive, so all enamel that is covered by a bracket should present a layer of adhesive on its surface.

The results of this study reveal that the greatest amount of adhesive remained on the teeth after debonding with the LODI, regardless of the type of bracket used. Considering the individual groups of brackets in the case of ceramic brackets, 84.36% of the adhesive remained after debonding with the debonding pliers, and 86.83% remained after debonding with LODI. Similar outcomes have been reported in other studies, in which most of the adhesive that remained after debonding with the debonding pliers was present on the tooth [4,5,18,19]. After debonding with the Weingart pliers, just over half of the adhesive (56.85%) remained on the enamel, which suggests that this method of debonding is the least preferable in the case of ceramic brackets.

**Figure 3:**
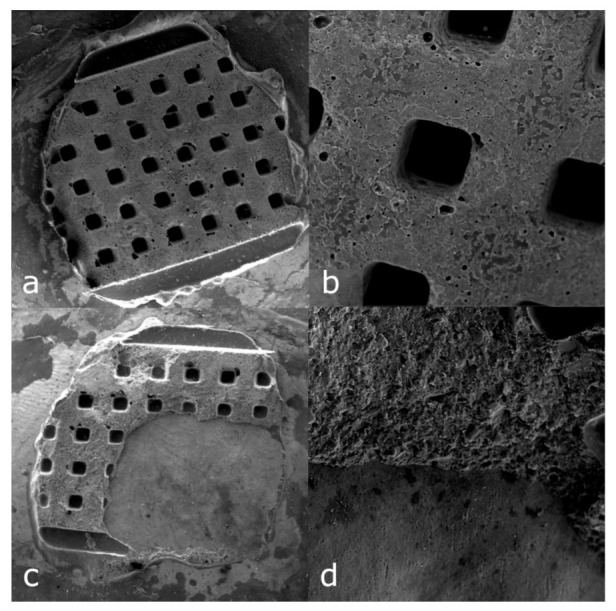
Comparison of the surface of the adhesive remaining on the enamel after Cannon bracket removal with LODI ((**a**) 15× magnification (**b**) 100× magnification) and with debonding pliers ((**c**) 15× magnification (**d**) 100× magnification).

The most suitable debonding method for brackets with anchor pylons bases is the use of LODI or Weingart pliers. The debonding pliers mainly caused failure at the enamel–adhesive interface, which theoretically poses a threat to enamel integrity. Figure 3a,b shows the surface of the adhesive remaining on the enamel after Cannon bracket removal with the LODI under 15× and 100× magnification, which presents the plain and intact structure of the material. Figure 3c,d depicts the sample after Cannon bracket removal with the debonding pliers. The second picture shows failure within the adhesive and the exposed surface of the enamel.

In the case of the brackets with a foil mesh base, the LODI caused more favorable bond separation compared to the Weingart pliers and the bracket removal pliers. Other studies have confirmed that LODI produces the most consistent separation at the bracket-adhesive interface, while the debonding of foil mesh brackets with bracket removal pliers inserted at the adhesive level resulted in a breakage line located close to the enamel [15,20]. Similar findings were described in a study comparing tensile and shear forces. The authors suggested that the bracket-cement interface is more resistant to compressive stress than it is to tensile stress [21]. This is in contrast with the results of Zarrinnia et al. [22], who found that bracket removal pliers showed separation at the bracket-adhesive level in all samples (*n* = 6). This can be explained by the application of different types of force: shear force was applied at the adhesive level in this study, while pulling force on the bracket wings was used in Zarrinnia et al.’s study. The amount of adhesive remaining after the debonding of brackets with an integral base by means of bracket removal pliers compared to foil mesh brackets was significantly lower, which was in accordance with the results of our previous study [8] and confirms that greater shear bond strength was connected with the proximity of the site of failure to the enamel.

This study also aimed to compare the results obtained by surface measurement and by survey of the ARI index. ARI scores are derived through subjective assessment (visual inspection of the enamel) and may, as some authors suggest, oversimplify the complex problem of bond failure [23]. The results of this study confirm that the ARI scale is reliable in most cases and derives data that are comparable to more time-consuming surface measurements. Some inconsistency of results was found only in borderline cases when the level of significance was close to the threshold value accepted for the purpose of the study (*p* < 0.05). Each metal bracket debonded with the LODI remained structurally intact, regardless of the architecture of its base. This is consistent with other studies and indicates that this method should be chosen when metal bracket rebonding is necessary [15,20,24]. Furthermore, a study that compared the level of discomfort reported by patients during the removal of orthodontic metallic brackets performed with four different debonding instruments found that the LODI group demonstrated the lowest pain scores [25].

In the case of the brackets with anchor pylons, the debonding pliers showed similar outcomes: all the Cannon brackets were classified as reusable, but as many as 80% of brackets debonded with Weingart pliers presented slot deformation (*n* = 13) or wing loss (*n* = 3). This divergence can be explained by the thickness and rigidity of the integral base of the Cannon brackets, which is resistant to shear force acting on the adhesive level, and the fragility of its wings, which can be easily deflected by squeezing. Quite different results were found in the mesh brackets groups, in which 55% of samples debonded with the debonding pliers demonstrated deformation of the bracket base, and 90% of the brackets separated with the Weingart pliers demonstrated slot distortion that prevented archwire insertion. This can be attributed to the architecture of the mesh brackets base, which is more prone to deflection compared to the integral base.

According to the manufacturer’s manual, debonding instruments cannot be used when the debracketing of ceramic brackets is planned. Although statistical tests showed no significant difference between different methods of ceramic bracket debonding, a pulling force exerted on the brackets’ wings resulted in the loss of wings in 10% of the samples (*n* = 2) and led to a breach of enamel integrity in a further 10% of the samples (*n* = 2). The results presented by Stumpf et al. suggest that the tensile strengths of metal and ceramic brackets are similar [26], but it is also known that the atomic structure of ceramic brackets accounts for their greatest fault-brittleness. Given that not only the manner of loading orthodontic brackets but also the bracket material affect the development of bond strength and stresses both on adhesive and enamel [27], further studies are required to assess the risk connected with the application of LODI to ceramic bracket debonding.

The findings of this study have to be seen in the light of some limitations. One of them is the different positioning of the debonding pliers in the case of the Cannon brackets, where, due to the long gingival tie wing, the pliers were placed obliquely. Dalaile et al. [13] found that oblique placement of the debonding pliers resulted in smaller ARI scores compared to occluso-gingival method, which is consistent with the results of our study. On the other hand, Delaile et al. applied different force systems (squeezing when pliers were placed obliquely and peeling off when pliers were positioned occluso-gingivally) and the exact comparison between these two studies is not accurate.

Another limitation concerns the random bonding of brackets to the buccal or lingual surfaces of third molars. Although in the case of any doubts the contact between the bracket base and the enamel surface was checked manually during the initial microscopic evaluation of the teeth, the positioning of the brackets on the lingual and buccal surfaces might have influenced the final result.

## 5. Conclusions

1. There was no difference between the different methods of debonding in terms of enamel damage, regardless of the bracket type.
2. LODI and Weingart pliers are considered to be the safest methods of debonding brackets with an integral base, and LODI is the safest method of debonding brackets with foil mesh due to bond failure at the bracket–adhesive interface.
3. Bracket removal pliers are considered to be the safest method for ceramic bracket debonding without damage to the bracket’s structure.
4. LODI is the recommended method when the rebracketing of metal brackets is planned.

## Figures and Tables

**Table 1:** Presence of enamel damage after bracket debonding.

Enamel Damage	Backlight Brackets			
	BRP (N = 20)	LODI (N = 20)	W (N = 20)	*p*
Yes	0 (0%)	2 (10%)	1 (5%)	*p* = 0.766
No	20 (100%)	18 (90%)	19 (95%)	
	**Cannon Brackets**			
	**BRP (N = 20)**	**LODI (N = 20)**	**W (N = 20)**	***p***
Yes	0 (0%)	0 (0%)	0 (0%)	*p* = 1
No	20 (100%)	20 (100%)	20 (100%)	
	**Victory (3M) Brackets**			
	**BRP (N = 20)**	**LODI (N = 20)**	**W (N = 20)**	***p***
Yes	1 (5%)	0 (0%)	2 (10%)	*p* = 0.766
No	19 (95%)	20 (100%)	18 (90%)	

*p*: Fisher’s exact test; BRP: bracket removal pliers; W: Weingart pliers.

**Table 2:** Comparison of the amount of adhesive remaining on the enamel after debonding according to the debonding method.

Adhesive Remnant Surface [%]	Backlight Brackets			*p*
	BRP (N = 20)	LODI (N = 20)	W (N = 20)	
mean ± SD	84.36 ± 19.74	86.83 ± 6.76	56.85 ± 30.98	*p* = 0.01 LODI, BRP > W
median	90.35	87.5	66.42	
quartiles	83.23–94.44	83.48–90.15	28.4–82.25	
**Adhesive remnant surface [%]**	**Cannon Brackets**			***p***
	**BRP (N = 20)**	**LODI (N = 20)**	**W (N = 20)**	
mean ± SD	25.46 ± 18.36	70.75 ± 33.75	61.58 ± 30.18	*p* < 0.01 LODI, W > BRP
median	22.23	91.86	64.95	
quartiles	15.95–32.91	43.7–97.89	33.91–89.8	
**Adhesive remnant surface [%]**	**Victory (3M) Brackets**			***p***
	**BRP (N = 20)**	**LODI (N = 20)**	**W (N = 20)**	
mean ± SD	55.89 ± 37.21	73.22 ± 28.75	34.21 ± 40.93	*p* = 0.017 LODI > W
median	57.11	84	9.06	
quartiles	22.11–96.19	60.92–95.19	0–70.03	

*p*: Kruskal-Wallis test followed by Dunn’s post hoc test; SD: standard deviation; BRP: bracket removal pliers; W: Weingart pliers.

**Table 3:** Comparison of the amount of adhesive remaining on the enamel after debonding according to the type of bracket used.

Adhesive Remnant Surface [%]	Bracket Removal Pliers			*p*
	Backlight (N = 20)	Cannon (N = 20)	Victory (N = 20)	
mean ± SD	84.36 ± 19.74	25.46 ± 18.36	55.89 ± 37.21	*p* < 0.01 Backlight > Victory > Cannon
median	90.35	22.23	57.11	
quartiles	83.23–94.44	15.95–32.91	22.11–96.19	
**Adhesive remnant surface [%]**	**LODI**			***p***
	**Backlight** **(N = 20)**	**Cannon** **(N = 20)**	**Victory** **(N = 20)**	
mean ± SD	86.83 ± 6.76	70.75 ± 33.75	73.22 ± 28.75	*p* = 0.852
median	87.5	91.86	84	
quartiles	83.48–90.15	43.7–97.89	60.92–95.19	
**Adhesive remnant surface [%]**	**Weingart pliers**			***p***
	**Backlight** **(N = 20)**	**Cannon** **(N = 20)**	**Victory** **(N = 20)**	
mean ± SD	56.85 ± 30.98	61.58 ± 30.18	34.21 ± 40.93	*p* = 0.036 Cannon > Victory
median	66.42	64.95	9.06	
quartiles	28.4–82.25	33.91–89.8	0–70.03	

*p*: Kruskal-Wallis test followed by Dunn’s post hoc test; SD: standard deviation.

**Table 4:** Comparison of ARI scores between groups according to the type of bracket used.

Debonding Method	Brackets Used	Sample Size	ARI				*p*-Value
			0	1	2	3	
**Bracket removal pliers**	Backlight	20	0 (0%)	1 (5%)	13 (65%)	6 (30%)	*p* < 0.01
	Cannon	20	2 (10%)	15 (75%)	3 (15%)	0 (0%)	
	Victory	20	0 (0%)	9 (45%)	9 (45%)	2 (10%)	
**LODI**	Backlight	20	0 (0%)	0 (0%)	16 (80%)	4 (20%)	*p* = 0.03
	Cannon	20	0 (0%)	6 (30%)	9 (45%)	5 (25%)	
	Victory	20	0 (0%)	5 (25%)	15 (75%)	0 (0%)	
**Weingart pliers**	Backlight	20	0 (0%)	9 (45%)	9 (45%)	2 (10%)	*p* = 0.04
	Cannon	20	0 (0%)	9 (45%)	9 (45%)	2 (10%)	
	Victory	20	6 (30%)	8 (40%)	4 (20%)	2 (10%)	

*p*: Fisher’s exact test.

**Table 5:** Comparison of ARI scores between groups according to the debonding method.

Brackets Used	Debonding Method	Sample Size	ARI				*p*-Value
			0	1	2	3	
**Backlight**	BRP	20	0 (0%)	1 (5%)	13 (65%)	6 (30%)	*p* = 0.01
	LODI	20	0 (0%)	0 (0%)	16 (80%)	4 (20%)	
	Weingart	20	0 (0%)	9 (45%)	9 (45%)	2 (10%)	
**Cannon**	BRP	20	2 (10%)	15 (0%)	3 (15%)	0 (0%)	*p* = 0.06
	LODI	20	0 (0%)	6 (30%)	9 (45%)	5 (25%)	
	Weingart	20	0 (0%)	9 (45%)	9 (45%)	2 (10%)	
**Victory**	BRP	20	0 (0%)	9 (45%)	9 (45%)	2 (10%)	*p* = 0.01
	LODI	20	0 (0%)	5 (25%)	15 (75%)	2 (10%)	
	Weingart	20	6 (30%)	8 (40%)	4 (20%)	2 (10%)	

*p*: Fisher’s exact test.

**Table 6:** Comparison of bracket reusability.

Bracket Condition	Backlight Brackets			*p*
	BRP (N = 20)	LODI (N = 20)	W (N = 20)	
Non-reusable	0 (0%)	2 (10%)	1 (5%)	*p* = 0.766
Reusable	20 (100%)	18 (90%)	19 (95%)	
**Bracket condition**	**Cannon brackets**			***p***
	**BRP (N = 20)**	**LODI (N = 20)**	**W (N = 20)**	
Non-reusable	0 (0%)	0 (0%)	16 (80%)	*p* < 0.01
Reusable	20 (100%)	20 (100%)	4 (20%)	
**Bracket condition**	**Victory brackets**			***p***
	**BRP (N = 20)**	**LODI (N = 20)**	**W (N = 20)**	
Non-reusable	11 (55%)	0 (0%)	18 (90%)	*p* < 0.01
Reusable	9 (45%)	20 (100%)	2 (10%)	

*p*: Fisher’s exact test (for Backlight group). *p*: chi-squared test (for Cannon and Victory groups).

## Data Availability

The datasets generated and analyzed during the current study are available in the Dropbox repository: https://www.dropbox.com/sh/o0rxw0937svi94p/AABdDhwyYBwUznFzfpZppMCga?dl=0 (accessed on 24 March 2021).
